# Novel CaO–SiO_2_–P_2_O_5_ Nanobioglass Activated with Hafnium Phthalocyanine

**DOI:** 10.3390/nano12101719

**Published:** 2022-05-18

**Authors:** Yuriy Gerasymchuk, Anna Wedzynska, Anna Lukowiak

**Affiliations:** Institute of Low Temperature and Structure Research, PAS, ul Okolna 2, 50-422 Wroclaw, Poland; y.gerasymchuk@intibs.pl (Y.G.); a.wedzynska@intibs.pl (A.W.)

**Keywords:** ternary glass, nanoparticles, dichlorometal-phthalocyanine, optical properties, medical applications

## Abstract

Bioactive glasses are materials which can be used in medicine for regeneration of hard and soft tissues. Their functionalization with active molecules or addition to composites broaden significantly the possible range of glass applications. Hereby, we describe photoactive nanoparticles of CaO–SiO_2_–P_2_O_5_ glass modified with dichlorohafnium (IV) phthalocyanine. The low-temperature, sol–gel based reverse micelle method was proposed for the synthesis, which allowed introduction of metal organic molecules into the glass composition. The morphology, structure, and composition of the material was described showing that spherical but agglomerated glass nanoparticles (size below 100 nm) were obtained in the ternary system. It was also shown that optical properties of the phthalocyanine complex were maintained after immobilization of the dye in the glass. The photoluminescence and generation of singlet oxygen molecules were observed under the light irradiation of the glass.

## 1. Introduction

Bioactive glass, glass-ceramic, and ceramic are examples of materials which can be used in medicine for reducing the wear and to amplify the duration of implants inside the human body or regeneration of hard and soft tissues, as well as for various applications in dentistry (dental restorative materials, mineralizing agents, coating material for dental implants, pulp capping, root canal treatment, and air-abrasion) [1,2,3,4]. Bioactive glasses are known of their excellent osteoconductivity, osteostimulation, and degradation rate. Although multi-component glasses obtained by the traditional melt-quenching method are widely used, they have a number of limitations, such as the need to use a high temperature (>1300 °C) during production and the lack of microporous structure inside the materials with a low specific surface area. Therefore, other techniques are used for bioactive glasses fabrication. Among them the sol–gel method is very promising [5,6].

The first reports of obtaining bioglass using the sol–gel reactions but performed at elevated temperature and pressure (solvothermal method) have been published recently [7,8,9]. Despite the synthesis carried out at a low temperature (150–220 °C), the final annealing of the materials took place at high temperature (above 500 °C), similar to the case of typical sol-gel process, which do not allow for modification of the glass with organic compounds.

The subject of this work was a development of a method suitable to produce nanometric ternary glass (SiO_2_–CaO–P_2_O_5_), where a low-temperature processing will allow functionalization with metal phthalocyanine complexes. Complexes of phthalocyanine (Pc) with metals (PcMe, where Me = Zn, Al, Si, Cu, Zr, etc.) are known as photoactive agents capable of generating singlet oxygen and reactive oxygen species under the light irradiation. Due to this activity, they are used as photosensitizers for photodynamic therapy [10,11] but also other medical applications of these compounds are currently under studies [12,13,14]. Fabrication of bioactive nanoglasses modified with PcMe might significantly broaden their application range. Despite the fact that an attempt to prepare bioactive silica-based structure activated with PcZn has been made [15], there is a lack of studies on SiO_2_–CaO–P_2_O_5_ with phthalocyanine complexes. Thus, we propose a new approach based on the sol–gel technology performed in the reverse microemulsion system, where nanosized calcium oxide and phosphoric acid are used as source of calcium and phosphorous oxides in the glass instead of commonly used calcium nitrate and triethyl phosphate, respectively. The synthesis condition allowed to introduce PcMe into the glass, which was proved by the optical spectroscopies.

## 2. Materials and Methods

### 2.1. Chemicals

Commercial reagents were purchased from Alfa-Aesar, Ward Hill, MA, USA and were used for synthesis without additional purification: HfCl_4_ (98%), 1,2-dicyanobenzene (phthalonitrile, 98%), 2-methylnaphtalene (97%), calcium nitrate tetrahydrate (97 + %), citric acid (99 + %), ethylene glycol (99%), Triton X100, 1-hexanol (99%), ammonium hydroxide (28%), tetraethoxysilane (99 + %), phosphoric acid (85% aq. soln.), and 1,3-diphenylisobenzofuran (97%, DPBF). The solvents used to wash or suspend the obtained materials were purchased from Avantor, Gliwice, Poland: benzene, toluene, methanol, ethanol (96%), acetone, and dimethyl sulfoxide (DMSO).

### 2.2. Synthesis of Glass and Its Components

Dichlorohafnium (IV) phthalocyanine complex (PcHf) was synthesized according to the method described in the previous reports [16,17]. Nanosized CaO used for glass fabrication was obtained from calcium nitrate. 5 g of Ca(NO_3_)_2_·4H_2_O was dissolved in 10 mL of deionized water. 10 g of citric acid and 5 mL of ethylene glycol were added to the solution under vigorous stirring on a magnetic stirrer. The reaction mixture was left on the magnetic stirrer for 2 h, then, it was transferred to a ceramic crucible and kept at 90 °C to form a gel. The gel was calcined at 700 °C for 2 h. The obtained CaO powder, after confirmation of its structure by X-ray diffractometry (XRD), was immediately used for further synthesis. More details about CaO can be found in the Supplementary Materials (Figure S1).

The method of obtaining the target product was developed on the basis of the fabrication of silica materials described by Zou and Chen [18] and by Gerasymchuk et al. [19]. The general procedure was the following: 0.5 g of CaO was dispersed in 18.5 mL of cyclohexane by ultra-sonification (UZDN M900-T (900 W, 22 kHz) disperser, Akadempribor, Sumy, Ukraine) and placed in 50 mL Erlenmeyer flask on a magnetic stirrer. Then, 1.5 mL of Triton^®^ X100 and 1.6 mL of 1-hexanol were added drop by drop and left under vigorous stirring for 15 min. 0.096 mL of H_3_PO_4_ in 1 mL of deionized water was added to the reaction mixture, which was further stirred for 15 min. 0.5 mmol of PcHf was dispersed in 5.2 mL of TEOS by ultra-sonification and added dropwise. After the next 15 min, 1.5 mL of ammonia was added to the reaction mixture, which was left on the magnetic stirrer for 5 h. Thereafter, the reaction flask was closed with Parafilm^®^ and placed in a refrigerator for 72 h. The product was precipitated with chilled acetone and separated using a centrifuge (6000 rpm, MPW M-Universal ultracentrifuge, Warsaw, Poland). The product was washed three times with ethanol, and three times with water followed by centrifugation, and dried at 90 °C. The received sample is marked as BGl@PcHfCl_2_.

### 2.3. Structure, Morphology and Photoactivity Investigations

X-ray diffractograms were recorded on a powder X’Pert Pro diffractometer by PANalitycal, United Kingdom. Scanning electron microscopy images were obtained from the probe deposited from methanol suspension on graphite plates with a field emission scanning electron microscope (FESEM, FEI Nova NanoSEM 230, Hillsboro, OR, USA) allowing to perform energy-dispersive X-ray spectroscopy (EDX) measurements. The grain size distribution has been determined with the ImageJ software (Wayne Rasband, National Institutes of Health, Bethesda, MD, USA) for about 150 particles observed on the SEM images. FTIR spectrum was recorded with a FT-IR spectrometer Biorad 575C. The probes were prepared by grinding of the investigated material with KBr and forming a pellet with a hand press. Optical properties were measured with Agilent CARY 5000 UV–Vis–NIR spectrometer (Braun, Germany) and Spectrophotometer FLS980 Edinburgh Instruments (Livingston, UK) equipped with a xenon lamp. The glass suspensions for measurement was prepared by ultra-sonification of 10 mg of material in 5 mL of DMSO or water. To verify the ability of the material to generate strongly reactive singlet oxygen molecules, the test with 1,3-diphenylisobenzofuran was performed. The absorption spectra were recorded for DPBF in DMSO in the presence of glass nanoparticles irradiated with a 150 W lamp (Philips) emitting light in the range of 550–900 nm. The light exposition varied between 10 s and 5 min. Zeta potential of the aqueous suspension of particles was measured using the a Zetasizer Nano ZS (Malvern Instruments, Malvern, Worcestershire, UK).

## 3. Results and Discussion

In the synthesis procedure proposed here, the reaction takes place in a reverse microemulsion system which is well known for fabrication of nanosized and monodispersed oxide particles in the sol–gel process [20]. TEOS, CaO, and H_3_PO_4_ are the precursors for silicon, calcium, and phosphorous oxides, respectively. In the microemulsion, all hydrophilic components of the mixture are enclosed in aqueous droplets surrounded by an oil phase. The hydrophobic tetraethoxysilane molecules (partially hydrolyzed at the beginning of synthesis in the presence of the orthophosphoric acid) penetrate the droplets through the surfactant interface layer, and the hydrolysis reactions proceed rapidly after addition of ammonia [21]. The resulting hydrophilic Si(OH)_4_ interacts with the CaO nanoparticles. At the same time CaO undergoes reaction with water giving Ca(OH)_2_ that also take part in the final glass particles formation. Moreover, TEOS is a good solvent for the hydrophobic PcHfCl_2_, and thus, the phthalocyanine is present in the reaction mixture and can be trapped in the formed silica-based matrix.

**Figure 1:**
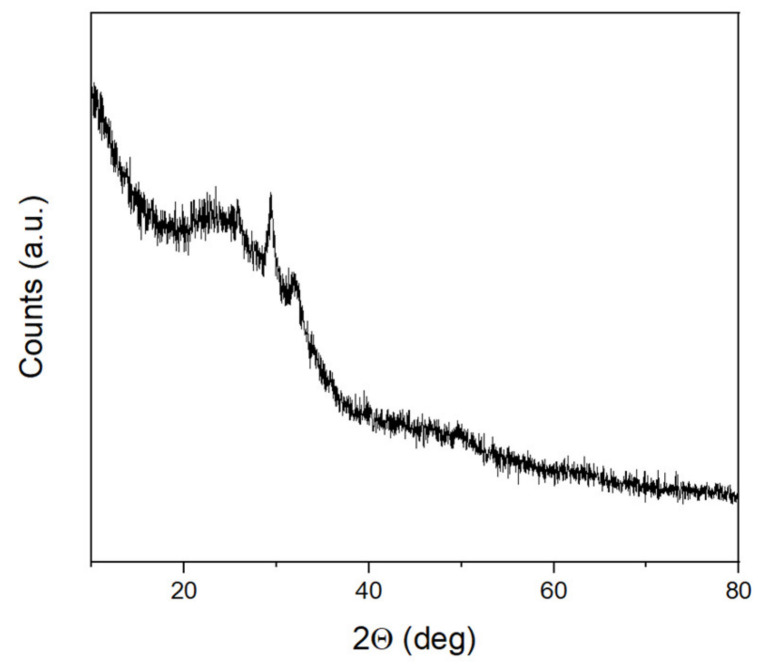
X-ray diffraction pattern of the obtained BGl@PcHfCl_2_.

The XRD pattern of the CaO–SiO_2_–P_2_O_5_ glass modified with the phthalocyanine complex showed an amorphous structure with an addition of crystalline phase assigned to the calcium carbonate (Figure 1). This would indicate that part of the Ca(OH)_2_ formed during synthesis has not entered the amorphous glass network and transformed partially into the crystalline phase of CaCO_3_ after reaction with CO_2_ from the air.

**Figure 2:**
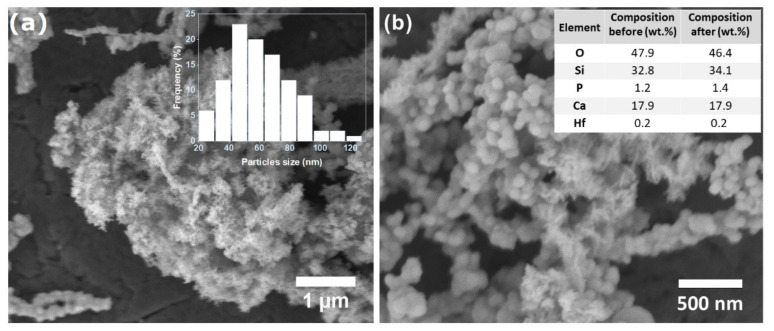
Representative SEM images of BGl@PcHfCl_2_. Insets show histogram of size particle distribution (**a**) and initial and final (based on EDX analysis) glass composition (**b**).

As observed in the SEM images, the glass was formed of small and spherical particles (Figure 2 and Figure S2). The mean size of particles was equal to 63 nm (SD = 23 nm). Zeta potential measured in water (−4.7 eV) pointed that powder do not form stable colloids. As seen in the SEM image as well, the particles forms agglomerates and aggregates (similar to the chain structures in Figure 2a).

The reagents taken for the synthesis were chosen in the amount which should provide the glass composition of 70SiO_2_–25CaO–5P_2_O_5_ (in wt.%). The elemental glass analysis (based on EDX measurements, Figure 2b) showed only small variation in the final glass composition. It indicated also the presence of hafnium that is introduced in the glass with the phthalocyanine complex. The content of PcHf in the glass would be equal to 0.8 mol.%.

**Figure 3:**
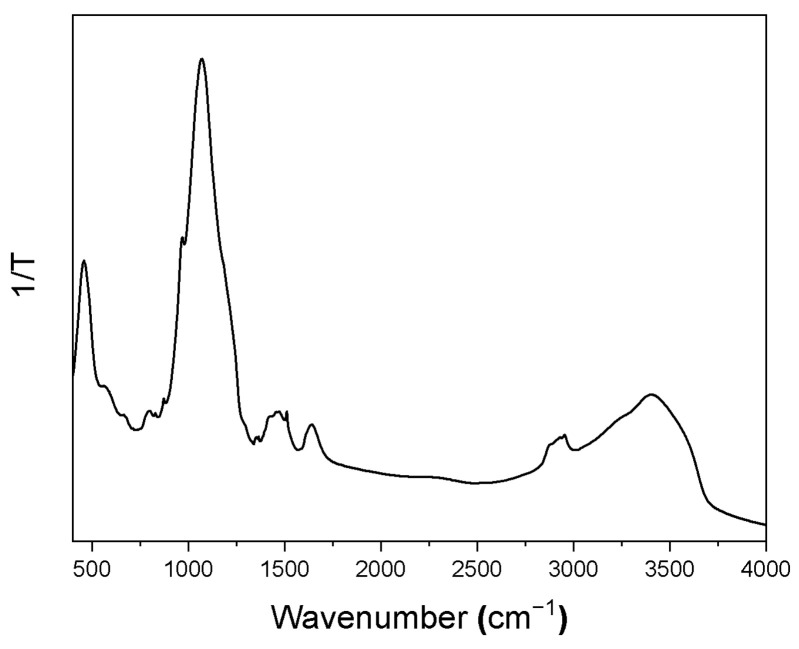
FTIR spectrum of BGl@PcHfCl_2_.

The FTIR spectrum (Figure 3) confirmed the formation of silica-based system [22,23,24,25,26]. The strong bands at 1076 and 1093 cm^−1^ are assigned to the symmetric and asymmetric stretching modes of the SiO_4_ tetrahedra, respectively, whereas the weak band at 797 cm^−1^ is caused by the vibration of the Si–OH group. The wide band between 3383 and 3463 cm^−1^ is due to the stretching vibration of the O–H bond from the silanol (Si–OH) groups and the HO–H vibration of the adsorbed water molecules. The small band at 1631 cm^−1^ is attributed to the flexural H–OH bond of adsorbed water molecules. Additional very weak bands in the spectrum confirm the presence of the PcMe molecules (see Table 1, Figure S3).

**Figure 4:**
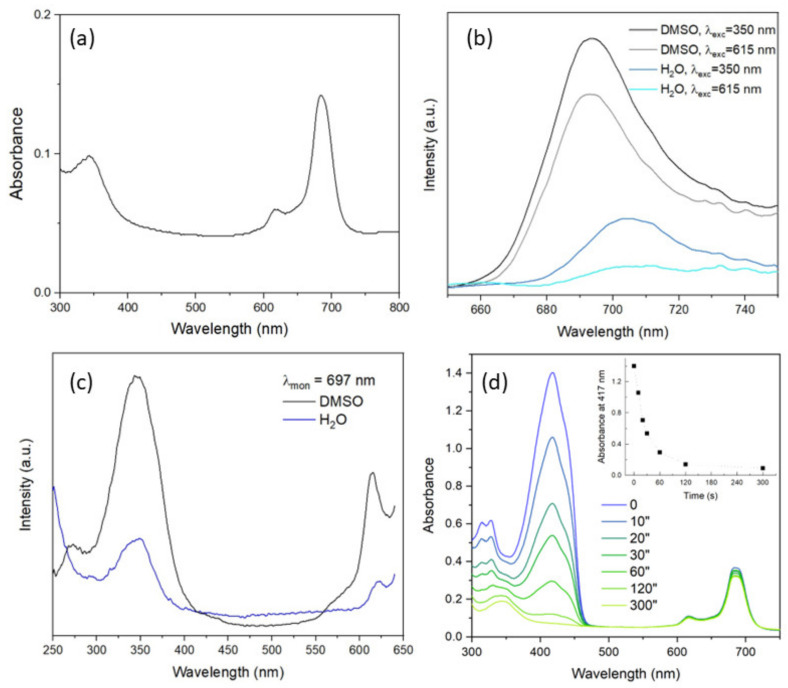
(**a**) Absorption spectrum of glass (BGl@PcHfCl_2_) in DMSO suspension. (**b**) Photoluminescence spectra of glass in DMSO and water under UV and red-light excitation. (**c**) Photoluminescence excitation spectra of glass in DMSO and water. (**d**) Absorption spectra of glass in DMSO in presence of diphenylisobenzofuran after different time of red-light irradiation (inset shows time-dependent decay of absorption band of DPBF monitored at 417 nm).

The absorption spectrum of the investigated glass suspended in dimethyl sulfoxide showed absorption bands originating from the molecules of hafnium (IV) phthalocyanine complex. The characteristic B and Q bands of PcHf were located at 343 nm and 685 nm, respectively (Figure 4a). Similar spectrum was observed for glass in water. These two bands can be used to excite the PcHf molecules. Additionally, indeed, the broad photoluminescence in the red/near-infrared region was observed for the sample in DMSO when excitation at 350 and 615 nm was used (Figure 4b). The bands intensity was weaker and red-shifted (704 vs. 694 nm) when the glass was suspended in water (the influence of the solvent on the photoluminescent properties of Pcs is well known [27,28,29,30]). Finally, the photoluminescence excitation spectra (Figure 4c) confirmed the possibility to activate PcHf using B and Q bands excitations.

Absorption spectroscopy was also used to verify photoactivity of the sample. 1,3-diphenylisobenzofuran was used as an indicator of singlet oxygen generation. The dye under the action of some reactive oxygen species is rapidly transformed to colorless 1,2-dibenzoylbenzene, that is observed as a decreasing intensity of an absorption band centered at 417 nm. Such an effect was obvious when BGl@PcHfCl_2_ in DMSO was irradiated with red lamp in the presence of DPBF (Figure 4d). The yellow color of the solution disappeared after 5 min showing that singlet oxygen is generated. At the same time, one may note slight variation in the intensity of the absorption band of PcHfCl_2_. That could indicate that the complex is not fully stable in the investigated system. The origin of the changes can be related to the nature of the PcHfCl_2_ itself, influence of solvent molecules, pH variation, photobleaching, or other effects.

## 4. Conclusions

The sol–gel method was used to prepare photoactive nanoparticles of CaO–SiO_2_–P_2_O_5_ glass modified with dichlorohafnium (IV) phthalocyanine. To maintain the complex in the glass, a low-temperature process was proposed, where the synthesis was performed in the reverse microemulsion system. Small glass nanoparticles were obtained having mean diameter of 63 nm. The sample had amorphous structure with small fraction of calcium carbonate. The optical properties of the glass (including photoluminescence and generation of reactive oxygen species) showed that the phthalocyanine complex is still active after immobilization in the glass. The proposed material which possess both photo- and bioactivity might be very promising for medical applications. For example, it could be used as an admixture to dental cements or wound dressings [4,31]. In comparison to standard bioactive glasses, its advantage is photoactivity. Thus, it would combine two functions—regeneration of tissues (through the formation of an inorganic hydroxyapatite-based matrix as in the case of bones and teeth) and photodynamic antibacterial treatment under the influence of red light (through the generation of singlet oxygen).

## Figures and Tables

**Table 1:** FTIR spectrum signal assignment.

Signal Maximum (Signal Range) cm^−1^	Vibration Assignment	Origin
3400 (3000–3680) vw	stretching vibration of O–H bond from the silanol (Si–OH) groups and HO–H vibration of adsorbed water molecules	Glass
1631 w	flexural H–OH bond of adsorbed water molecules	Glass
1511 vw	ν(CNC) + ν(φ) + δ(CH)	Pc macrocycle
1220 sh	PO^2–^ asymmetric/P = O stretching	Glass
1090 sh	asymmetric stretching modes of SiO_4_ tetrahedra	Glass
1076 vs	symmetric stretching modes of SiO4 tetrahedra	Glass
962 (900–980) sh	υ_3_–Si–O stretching/PO_4_^3−^ groups	Glass
969 vw	δ(CH) + δ(φ) + ρ(MeN_4_)	Pc macrocycle + coordinated metal
869 vw	γ(CH) + δ(φ) + δ(CNC) + δ(CN)	Pc macrocycle
797 vw	Si–OH group	Glass
611 vw	asymmetric stretching vibrations of PO_4_^3−^	Glass
564 w	asymmetric stretching vibrations of PO_4_^3−^/υ_4_–P–O bending mode	Glass
467 vw	Si–O–Si rocking/Si–O–Si symmetric bending mode	Glass
446 sh	Si–O–Si rocking/Si–O–Si symmetric bending mode	Glass

## Data Availability

The data presented in this study are available on request from the corresponding author.

## Supplementary Materials

The following supporting information can be downloaded at: https://www.mdpi.com/article/10.3390/nano12101719/s1, Figure S1: X-ray diffraction pattern of the obtained CaO powder.; Figure S2: SEM image showing diameters of selected nanoparticles.; Figure S3: IR spectrum of dichlorohafnium (IV) phthalocyanine.
