# Supplementary material for: Novel CaO–SiO2–P2O5 Nanobioglass Activated with Hafnium Phthalocyanine
Source: Nanomaterials (Basel). 2022 May 18;12(10):1719. doi: 10.3390/nano12101719 (PMC9146838; doi:10.3390/nano12101719)
Supplement: Supplementary file 1 [file nanomaterials-12-01719-s001.zip › nanomaterials-1699060-Supplementary.pdf]

nanomaterials

Supplementary Materials

Novel CaO–SiO_2–P_2O_5 Nanobioglass Activated with Hafnium Phthalocyanine

Yuriy Gerasymchuk, Anna Wedzynska and Anna Lukowiak *

Institute of Low Temperature and Structure Research, PAS, ul Okolna 2, 50-422 Wroclaw, Poland; y.gerasymchuk@intibs.pl (Y.G.); a.wedzynska@intibs.pl (A.W.)
* Correspondence: a.lukowiak@intibs.pl

The obtained calcium oxide, which was used as a source of calcium needed in the glass synthesis, was characterized using the X-ray diffraction analysis. The XRD pattern of the powder is shown in Figure S1. The Scherrer equation

$$\boldsymbol{D} = \mathbf{K}\boldsymbol{\lambda}/(\boldsymbol{\beta}\boldsymbol{cos\theta})$$

(where D is the mean size of the grain size, K is a dimensionless shape factor (0.9), λ is the X-ray wavelength (Cu Kα, 1.5406 Å), β is the line broadening at half the maximum intensity (FWHM). θ is the Bragg angle), was used to calculate the size of the crystallites showing that the particles had diameter of 40 nm.

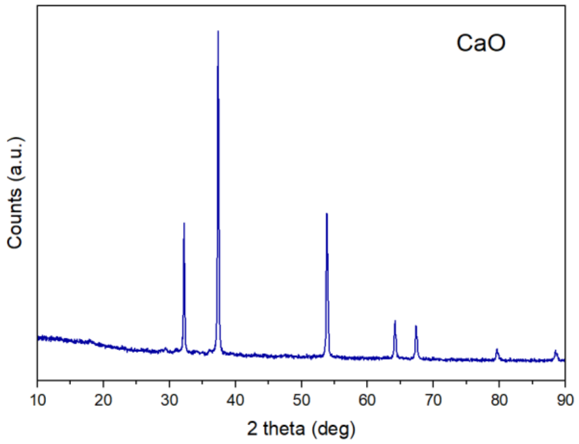

Figure S1. X-ray diffraction pattern of the obtained CaO powder.

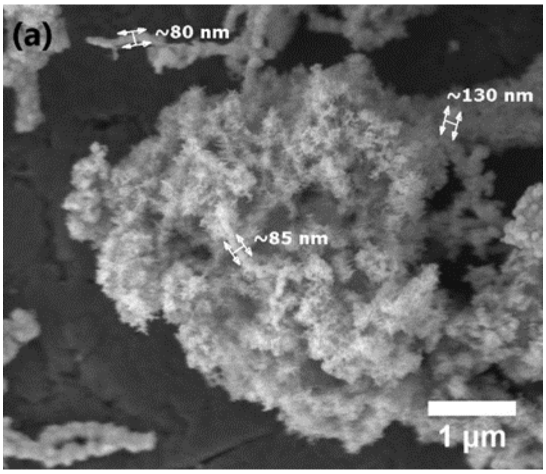

Figure S2. SEM image showing diameters of selected nanoparticles.

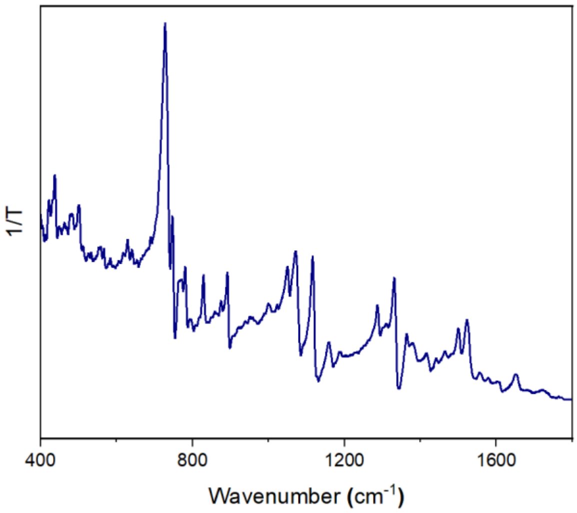

Figure S3. IR spectrum of dichlorohafnium(IV) phthalocyanine.
